# Meetings of Societies

**Published:** 1895-03

**Authors:** 


### MEETINGS OF SOCIETIES.

### Bristol Medico-Chirurgical Society.

*January 9th*, 1895.

Dr. A. J. Harrison, President, in the Chair.

Dr. B. M. H. Rogers opened a discussion on Diphtheria. He said that from a large number of difficult problems in the diagnosis, etiology and treatment of the disease, he had selected some half-dozen questions which he thought might be profitably considered. The disease he considered to be a local one, followed by constitutional infection, and he strongly advocated the elimination of the misleading word "croup" from medical nomenclature.

Some explanation of the almost universal increase in incidence and mortality from the disease was asked for, and the influence of large schools pointed out. The necessity of bacteriological examination as a help to diagnosis was insisted on, and the difficulties connected with the recognition of the bacillus from that found in pseudo-membranous laryngitis referred to. Some suggestions were made to the probable habitat of the bacillus outside the human body, and the possibility of infection from animals as well as from milk noticed.

Respecting treatment, he referred to the results of American physicians on the value of local applications to the throat, which show that solutions strong enough to be germicidal are dangerous from their poisonous properties, and may cause extension of the disease from their irritating effects; while bland solutions, such as saline solutions, are of equal value and are not attended with danger. The practice of intubation in place of tracheotomy had not, he stated, found favour with English physicians; but there were certain points in its favour, and it should therefore be given further trial.

In conclusion, he referred to the new treatment of the disease by the immunised serum, and sketched briefly the theoretical reasons and the laboratory experience which had led Behring and Roux to recommend it, giving also a few statistics of the results obtained by its use in this country.

Dr. James Swain remarked that, speaking generally, he was opposed to intubation of the larynx in diphtheria, as he had on several occasions to perform a subsequent tracheotomy. The success of antitoxin might modify one's views, and where the dyspnœa is slow in onset and there is reason to believe the membrane has not passed beyond the larynx, intubation might be preferred; but where the membrane has reached the trachea and bronchi, and the dyspnœa has been rapidly progressive, then we shall be well advised in performing tracheotomy. The time when either of these operations should be performed must be left to individual judgment in each case; but there are two conditions which he regarded as distinct indications for operation: (1) Periods of calm alternating with paroxysmal attacks of severe dyspnœa, for the calmness is that of exhaustion; (2) when there is a rapid and marked diminution of arterial pressure at the wrist during inspiration in association with the dyspnœa. He spoke in favour of local antiseptics and solvents, and, as the result of original experiments, he thought that the biniodide of mercury in combination with liquor potassæ was best. The use of pilocarpine hypodermically was dangerous.

Dr. W. Barrett Roué read notes of a case of diphtheria successfully treated by an injection of five cc. of antitoxin *after* tracheotomy had been performed. Seven days after the injection, the urine was free from albumen and the tracheotomy wound healed. The patient, a child four years of age, made a rapid and uninterrupted recovery. There were no sequelæ.

Dr. W. L. Christie said that the Bristol Hospital for Children and Women had since October received twelve cases notified as diphtheria. Only one failed to pass Mr. Stoddart's bacteriological test, but they all had the usual clinical accompaniments. Antitoxin was used in eight cases; in five, it was combined with tracheotomy. One, aged four, marred antitoxin results by dying of cardiac failure during tracheotomy three hours after admission, when six cc. had been administered. One, aged three, died of cardiac failure four hours after operation, despite five cc. twice repeated. Another, aged six, died on sixth day of diphtheritic broncho-pneumonia. Of the five recoveries, one had abscesses after serum two days uncorked but clear. There was also increased albuminuria from, he believed, elimination of the serum injected. One or two had paresis of soft palate, but no other complications arose. The dose used is from five to ten cc. (Ruffer), repeated daily if required, by a Debove's sterilisable syringe. The temperature fell if high, or rose if low; the pulse improved, the membrane loosened, and the toxæmia lessened. Alongside these, three were treated *without* antitoxin. One, aged three, mild at first, nearly died of intense nervous prostration and submental cellulitis from tonsillar coccic invasion. One, aged five, died on ninth day of cardiac failure. The tracheotomy wound became infected, and was dry, inflamed, and spreading. The bacilli were long, and prognosis therefore was bad. The other case, mild from the first, with short bacilli, recovered rapidly, as did the non-bacillary case. The treatment is well worth further trial, with of course *fresh* serum and *well-boiled* hypodermic apparatus.

Mr. F. W. Stoddart spoke at length on the bacteriology (see p. 18). He exhibited numerous cultures, and showed some photographs by means of the lantern.

Dr. H. L. Ormerod, during the past two years at the Bristol Royal Infirmary, had treated eighteen cases of diphtheria by intubation, sixteen of which required a subsequent tracheotomy; of the other two, one died from toxæmia and the other recovered. Of six cases treated by Aronson's antitoxin, two—each aged eleven years—made rapid recoveries, and the other four died: these four were, however, all cases in which tracheotomy was necessary, and were aged four and a-half years, three and a-half years, fourteen months and thirteen months.

Mr. T. Pagan Lowe claimed to have reduced the mortality of diphtheria in his practice to ten per cent. by the local application of alcohol, a result which compared very favourably with the nineteen per cent. mortality obtained with the antitoxin treatment. The alcohol is applied in the form of spirits of wine, brandy, or whiskey, either neat or diluted with an equal quantity of water. He advocated the early disinfection of the throat in all cases, and protective antitoxin injections to members of the same family who were not affected.

Dr. F. H. Edgeworth said that there were two classes of cases in which the difficulty of diagnosis was great: those of pharyngeal diphtheria in which there was no membranous exudation and no grave constitutional symptoms, but which, as shown by the subsequent occurrence of paralysis, were really diphtheritic; and those of primary laryngeal diphtheria, where no view of the larynx could be obtained and no membrane had been coughed up. In view of treatment by antitoxin serum, diagnosis was of no material importance in the first class, as there was no risk to life; but in the second it was, and he asked what degree of gradually oncoming pyrexial laryngeal obstruction in a previously healthy child would justify one in employing this remedy. Reference was made to cases illustrating these difficulties.

Mr. J. Ewens remarked that in an extensive country practice in which diphtheria was very common, he found treatment by chlorate of potash and iron, with local applications of liq. ferri perchlor., generally successful; and in no case seen early had the disease, when limited to the pharynx, spread to the larynx. In mild laryngeal complications, inhalation of tinct. iodi with hot water was very useful.

Dr. R. Shingleton Smith said that this discussion should not close without more reference to the local treatment of diphtheria. Prof. Loeffler's results were remarkable[1]: in ninety-six cases treated, three-fourths of which were shown by bacteriological examination to be true

[1] The composition of the fluid used is given on p. 25 of this number.

diphtheria, not a single death occurred. A mixture of guaiacol and glycerine had also been used recently (*Medical Week*, Jan. 4th, 1895). Probably a mixture of guaiacol with menthol in white oil would be found to give good results, or perhaps equal parts of tinct. iodi co. and guaiacol.

---

*February 13th*, 1895.

Dr. A. J. HARRISON, President, in the Chair.

Mr. A. W. PRICHARD gave the clinical record of a case of intestinal obstruction in a girl aged thirteen weeks. When the child was admitted into the Infirmary its abdomen was enormously distended. There had been no motion for three days. The abdomen was opened below the umbilicus. One thin-walled viscus filled the upper three-fourths of the abdominal cavity. This was tapped and then opened freely. Fæces and gas escaped. The wound was sewed to the parietes. For nine days the patient did well. Fæces passed freely from wound and anus. On ninth day death took place from peritonitis. The autopsy disclosed a congenital malformation which was described in a paper by Mr. H. F. Mole, in whose absence it was read by Dr. F. H. Edgeworth. The large intestine bifurcated two inches beyond the cæcum: one branch had the normal situation of the colon and ended in a rectum; the other branch, after a short course, opened into a large skin-lined sac, from which it was continued as a second tube, of constricted calibre, ending in a rectum lying in front of the other. The proctodeum opened into both recta. The other organs were normal. The sac was thought to be a dermoid cyst. Obstruction was probably brought about by distension of the sac, owing to the (congenital or acquired) narrowing of the intestine beyond it, with fæces and flatus, whereby the other large intestine was occluded. No similar case could be found recorded in medical literature.

Dr. J. E. PRICHARD exhibited the following specimens: (1) Portion of intestine of an infant five days old, in which there was a complete occlusion of the ileum a few inches above the ileo-cæcal valve. The site of the malformation was probably the point where the omphalo-mesenteric duct opens into the intestinal tract. (2) An anencephalous fœtus of about the eighth month. (3) Specimen of horse-shoe kidney.

Dr. A. E. AUST LAWRENCE brought forward two cases of complete inversion of the uterus. The first was caused by the placenta being dragged away by means of the cord. The inversion had existed three months. Taxis under chloroform failed to replace the uterus. It was reduced in thirty hours by constant elastic pressure with Aveling's repositor. The second case was produced by the dragging of a polypus growing from the fundus uteri, and which had become extruded beyond the vulva. The polypus was removed, and the uterus re-inverted itself in twenty-four hours.

Mr. J. EWENS showed a specimen of parovarian tumours (right and left sides) removed successfully from a woman, æt. forty-seven, who had been treated for four years for retroverted uterus, the interest attaching to the case being the multilocular character of the tumours. A hard swelling was felt behind the uterus, and a tumour reached to the umbilicus, with a somewhat indistinct though definite fluctuation. The ordinary operation was performed. The right tumour consisted of six cysts, several of which had to be tapped before it could be delivered. The ovary and Fallopian tube were removed. The left tumour contained four cysts, two of which being tapped, it shelled out of its bed, no pedicle existing. The fluid of all the cysts was clear.

*March 13th*, 1895.

Dr. A. J. Harrison, President, in the Chair.

The President spoke in appropriate terms of the loss the Society had sustained in the death of Dr. J. G. Davey, a Past President. A resolution of sympathy with the family was passed.

Dr. W. L. Christie showed a patient with asymmetrical development of cranium and lower jaw, with spinal symptoms; and another patient with peripheral neuritis, showing trophoneurotic changes.

Mr. J. Greig Smith exhibited: (1) Large sarcoma of breast. (2) Hydronephrotic kidney, due, he considered, to pressure on the ureter. The patient had extreme prolapse of the uterus. (3) Sub-phrenic abscess cavity, caused by a perforating ulcer of the stomach. (4) Malignant stricture at the ileo-cæcal valve. (5) Gall-stones from two recent operation cases. (6) Small calculus from the female bladder.

The President exhibited a specimen of necrosis of lower jaw in a Polar bear; and fracture of the base of skull in a Brahmin bull.

Dr. J. Michell Clarke read a paper, illustrated with numerous diagrams and specimens, on two cases of extra-dural pressure on spinal cord: (1) from inflammatory swelling in a case which presented symptoms of syringo-myelia; (2) from a localised tubercular mass in caries. Mr. Barclay described the operation he had performed in the first case, mentioning the great difficulty he had experienced in stopping the excessive hemorrhage.

Mr. J. Paul Bush read a paper on a case of foreign body in the bronchus of a child aged eight, and exhibited a steel hat-pin, over three inches in length, which had been expelled through the mouth three months after tracheotomy had been performed.

J. Paul Bush, *Hon. Sec.*

---

### Devon and Exeter Medico-Chirurgical Society.

*Annual Meeting, January 18th*, 1895.

Mr. E. J. Domville, President, in the Chair.

The Report of the Council was read. The following officers were elected for the ensuing year:—President; Mr. G. H. Johnson: Vice-president; Mr. J. D. Harris: Treasurer; Mr. J. D. Harris: Honorary Secretary; Dr. R. V. Solly: Members of Council; Mr. J. L. Callaghan, Dr. P. M. Deas, Dr. W. Gordon, Mr. F. Morgan, Mr. J. H. Potter, and Dr. J. R. Thomas: Auditors; Mr. R. Coombe and Mr. J. Mortimer.

Mr. A. C. Roper read notes of a case of rheumatic pericarditis and endocarditis with profound anæmia.

Mr. R. Coombe opened a discussion on the present position of antiseptics in surgery.

---

*February 15th*, 1895.

Mr. G. H. Johnson, President, in the Chair.

A vote of thanks was given to Dr. A. G. Blomfield, the retiring Secretary.

Dr. J. Raglan Thomas read a paper on infantile convulsions.

Mr. J. H. Potter read a paper on some points of diagnosis and treatment of venereal diseases.

R. V. Solly, *Hon. Sec.*

